# CPMCGLM: an R package for *p*-value adjustment when looking for an optimal transformation of a single explanatory variable in generalized linear models

**DOI:** 10.1186/s12874-019-0711-2

**Published:** 2019-04-16

**Authors:** Benoit Liquet, Jérémie Riou

**Affiliations:** 1 Université de Pau et Pays de l’Adour, UFR Sciences et Techniques de la Cote Basque-Anglet UMR CNRS 5142, Allée du Parc Montaury, Anglet, 64600 France; 2 ARC Centre of Excellence for Mathematical and Statistical Frontiers and School of Mathematical Sciences at Queensland University of Technology, Brisbane, Australia; 3 MINT UMR INSERM 1066, CNRS 6021, Université d’Angers, UFR Santé, 16 Boulevard Davier, Angers Cedex, 49085 France

**Keywords:** R package, Generalized linear model, Resampling, *p*-value adjustment, Multiple testing, Union intersection test, Optimal cutoff point determination

## Abstract

**Background:**

In medical research, explanatory continuous variables are frequently transformed or converted into categorical variables. If the coding is unknown, many tests can be used to identify the “optimal” transformation. This common process, involving the problems of multiple testing, requires a correction of the significance level.

Liquet and Commenges proposed an asymptotic correction of significance level in the context of generalized linear models (GLM) (Liquet and Commenges, Stat Probab Lett 71:33–38, 2005). This procedure has been developed for dichotomous and Box-Cox transformations. Furthermore, Liquet and Riou suggested the use of resampling methods to estimate the significance level for transformations into categorical variables with more than two levels (Liquet and Riou, BMC Med Res Methodol 13:75, 2013).

**Results:**

CPMCGLM provides to users both methods of *p*-value adjustment. Futhermore, they are available for a large set of transformations.

This paper aims to provide insight the user an overview of the methodological context, and explain in detail the use of the CPMCGLM R package through its application to a real epidemiological dataset.

**Conclusion:**

We present here the CPMCGLMR package providing efficient methods for the correction of type-I error rate in the context of generalized linear models. This is the first and the only available package in R providing such methods applied to this context.

This package is designed to help researchers, who work principally in the field of biostatistics and epidemiology, to analyze their data in the context of optimal cutoff point determination.

## Background

In applied statistics, statistical models are widely used to assess the relationship between an explanatory and a dependent variable. For instance, in epidemiology, it is common for a study to focus on one particular risk factor. Scientists may wish to determine whether the potential risk factor actually affects the risk of a disease, a biological trait, or another outcome. In this context, statisticians use regression models with an outcome *Y*, a risk factor *X* (continuous variable of interest) and *q*−1 adjustment variables. In clinical and psychological research, the usual approach involves dichotomizing the continuous variable, whereas, in epidemiological studies, it is more usual to create several categories or to perform continuous transformations [1]. It is important to note that the categorization of a continuous predictor can only be justified when threshold effects are suspected. Furthermore, when the assumption of linearity is found to be untenable, a fractional polynomial (FP) transformation should always be favoured.

For instance, let us consider a categorical transformation of *X*. When the optimal set of cutoff points is unknown, the subjectivity of the choice of this set may lead to the testing of more than one set of values, to find the “optimal” set. For each coding, the nullity of the coefficient associated with the new coded variable is tested. The coding finally selected is that associated with the smallest *p*-value. This practice implies multiple testing, and an adjustment of the *p*-value is therefore required. The CPMCGLM package [2] can be used to adjust the *p*-value in the context of generalized linear models (GLM).

We present here the statistical context, and the various codings available in this R package. We then briefly present the available methods for type-I error correction, before presenting an example based on the PAQUID cohort dataset.

## Implementation

### Statistical setting

#### Generalized linear model

Let us consider a generalized linear model with *q* explanatory variables [3], in which *Y*=(*Y*_1_,…,*Y*_*n*_) is observed and the *Y*_*i*_’s are all identically and independently distributed with a probability density function in the exponential family, defined as follows:

$$f_{Y_{i}}(Y_{i},\theta_{i},\phi)= exp \left \{ \frac{Y_{i}\theta_{i}-b(\theta_{i})}{a(\phi)} + c(Y_{i},\phi) \right \}; $$

with $\mathbb {E}[Y_{i}]=\mu _{i}=b'(\theta _{i}),\mathbb {V}ar[Y_{i}]=b''(\theta _{i})a(\phi)$ and where *a*(·),*b*(·), and *c*(·) are known and differentiable functions. *b*(·) is three times differentiable, and its first derivative *b*^′^(·) can be inverted. Parameters (*θ*_*i*_,*ϕ*) belong to $\Omega \subset \mathbb {R}^{2}$, where *θ*_*i*_ is the canonical parameter and *ϕ* is the dispersion parameter. The CPMCGLM package allows the use of linear, Poisson, logit and probit models. The specifications of the model are defined with formula, family and link arguments, as a glm() function.

In this context, the main goal is evaluating the association between the outcome *Y*_*i*_ and an explanatory variable of interest *X*_*i*_, adjusted on a vector of explanatory variables *Z*_*i*_. The form of the effect of *X*_*i*_ is unknown, so we may consider *K* transformations of this variable *X*_*i*_**(k)**=*g*_*k*_(*X*_*i*_) with *k*=1,…,*K*.

For instance, if we transform a continuous variable into a categorical variable with *m*_*k*_ classes, then *m*_*k*_−1 dummy variables are defined from the function *g*_*k*_(·): $\mathbf {X_{i}(k)}=g_{k}(X_{i})=\left (X_{i}^{1}(k),\hdots,X_{i}^{m_{k}-1}(k)\right)$. *m*_*k*_ different levels of the categorical transformation are possible.

The model for one transformation *k* can be obtained by modeling the canonical parameter *θ*_*i*_ as:

$$\theta_{i}(X,Z,k)=\boldsymbol{\gamma} \mathbf{Z_{i}}+ \boldsymbol{\beta_{k}} \mathbf{X_{i}(k)},\ 1 \le i \le n;$$

where $\mathbf {Z_{i}}=\left (1,Z_{i}^{1},\hdots,Z_{i}^{q-1}\right), \boldsymbol {\gamma }=(\gamma _{0},\hdots,\gamma _{q-1})^{T}$ is a vector of *q* regression coefficients, and ***β***_***k***_ is the vector of coefficients associated with the transformation *k* of the variable *X*_*i*_.

#### Multiple testing problem

We consider the problem of testing

$$\mathscr{H}_{0,k}: \boldsymbol{\beta_{k}} = 0 \:\: \text{ against} \:\: \mathscr{H}_{1,k}: \boldsymbol{\beta_{k}} \neq 0, $$

simultenaously for all *k*∈{1,…,*K*}. For each transformation *k*, one test score *T*_*k*_(*Y*) is obtained for the nullity of the vector ***β***_***k***_ [4]. We ultimately obtain a vector of statistics **T**=(*T*_1_(*Y*),…,*T*_*K*_(*Y*)). Introduce the associated *p*-value as

$$p_{k}(y) = \mathbb P_{\boldsymbol{\beta_{k}} = 0}(|T_{k}(Y)|\ge|T_{k}(y)|), \:\: 1\le k \le K, $$

where *y* is the realization of *Y*.

### Significance level correction

To cope with the multiplicity problem, we aim at testing [5]:

$$\mathscr{H}_{0} \: : \: \bigcap_{k=1}^{K} \mathscr{H}_{0,k} \:\: \ \text{against} \:\: \mathscr{H}_{1} \: : \: \bigcup_{k=1}^{K} \mathscr{H}_{1,k}, $$

by which we mean that *X* has an effect on *Y* if and only if at least one transformation of *X* has an effect on *Y*. A natural approach is then to consider the maximum of the individual test statistics *T*_*k*_(*Y*), or, equivalently, the minimum of the individual *p*-values *p*_*k*_(*Y*), leading to the following *p*-values:

$$p^{maxT}(y) = \mathbb P_{Y\sim P_{0}} \left(T^{maxT}(Y) \ge T^{maxT}(y) \right), $$

where *P*_0_ denote the distribution of *Y* under the null and *T*^*maxT*^(·)=max1≤*k*≤*K*{|*T*_*k*_(·)|}, or

$$p^{minP}(y) = \mathbb P_{Y\sim P_{0}} \left(p^{minP}(Y) \le p^{minP}(y) \right), $$

where *p*^*minP*^(·)=min1≤*k*≤*K*{*p*_*k*_(·)}.

Moreover, if *X* has an effect on *Y* (e.g. $\mathscr {H}_{0}$ is rejected), the best coding corresponds to the transformation *k* which obtains the highest individual test statistic realization *T*_*k*_(*y*), or, equivalently, the smallest individual *p*-value realization *p*_*k*_(*y*).

#### Bonferroni method

The first method available in this package is the Bonferroni method. This is the most widely used correction method in applied statistics. It has been described by several authors in various applications [6–10]. The Bonferroni method rejects $\mathscr {H}_{0}$ at level *α*∈[0,1] if

1 $$ p^{minP}(y) \le \frac{\alpha}{K},  $$

where *K* is related to the total number of tests performed by the user. However, this method is conservative, particularly when the correlation between test results is high and the number of transformations is high.

#### Exact method

The second method proposed in this package is the asymptotic exact correction developed by Liquet and Commenges for generalized linear models [11, 12]. This method is valid only for binary transformations, fractional polynomial transformations with one degree (i.e. FP1) and Box-Cox transformations. It is based on the joint asymptotic distribution of the test statistics under the null. Indeed, the *p*-value *p*^*maxT*^ can be calculated as follows:

$$\begin{array}{@{}rcl@{}} p^{maxT}(y) &=& 1 - \mathbb{P}_{Y\sim P_{0}}\left(T^{maxT}(Y) < T^{maxT}(y) \right) \\ &=& 1 - \mathbb{P}_{Y\sim P_{0}} (T_{1}(Y)<T^{maxT}(y); \hdots ;\\ && T_{K}(Y)< T^{maxT}(y)). \end{array} $$

We then calculated the probability $\mathbb {P}_{Y\sim P_{0}} \big (T_{1}(Y)<T^{maxT}(y); \hdots ; T_{K}(Y)< T^{maxT}(y)\big)$ by numerical integration of the multivariate Gaussian density (e.g., the asymptotic joint distribution of (*T*_*k*_)_1≤*k*≤*K*_). Several programs have been written to solve this multiple integral. In this package, we used the method developed by Genz and Bretz in 2009 [13], available in the mvtnorm R package [14].

#### Minimum *p*-value procedure

The approach based on *p*^*minP*^, called the minimum *p*-value procedure, allows to combine statistical tests for different distributions. It is therefore possible to combine dichotomous, Box-Cox, fractional polynomial and transformations into categorical variables with more than two levels. However, the distribution of *p*^*minP*^ is unknown and we use resampling-based methods. These procedures take into account the dependence structure of the tests for evaluation of the significance level of the minimum *p*-value procedure. These procedures can therefore be used for all kinds of coding.

##### Permutation test procedure

The first resampling-based method is a permutation test procedure. This procedure is used to build the reference distribution of statistical tests based on permutations. From a theoretical point of view, the statistical test procedures are developed by considering the null hypothesis to be true, i.e. in our context, under the null hypothesis, *X*_*i*_ has no impact on *Y*. Under the null hypothesis, if the exchangeability assumption is satisfied [15–20], then resampling can be performed based on the permutation of *X*_*i*_ the variable of interest in our dataset. The procedure proposed by Liquet and Riou could be summarized by the following algorithm [6]:

1. Apply the minimum *p*-value procedure to the original data for the *K* transformations considered. We note *p*_*min*_ the realization of the minimum of the *p*-value;
2. Under $\mathscr {H}_{0,k}$, *X*_*i*_ has no effect on the response variable *Y*, and a new dataset is generated by permuting the *X*_*i*_ variable in the initial dataset. This procedure is illustrated in the following Fig. 1;

**Fig. 1:**
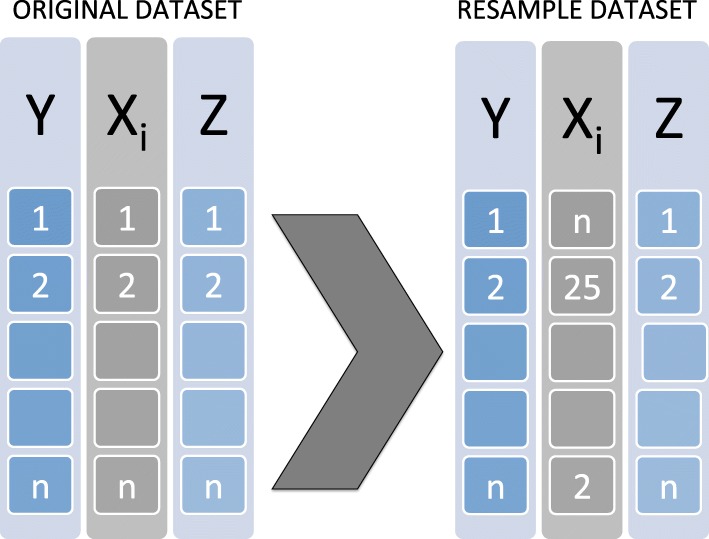
Permutation Principle under the null hypothesis $\left (\mathscr {H}_{0,k}\right)$
3. Generate *B* new datasets $s^{*}_{b}$, *b*={1,...,*B*} by repeating step 2 *B* times;
4. For each new dataset, apply the minimum *p*-value procedure for the transformation considered. We note $p^{*b}_{\text {min}}$ the smallest *p*-value for each new dataset.
5. The *p*-value is then approximated by:
  $$\widehat{p^{minP}}=\frac{1}{B}\sum_{b=1}^{B}I_{\left\{p_{\text{min}}^{*b} < p_{\text{min}}\right\}},$$
  where *I*_{·}_ is an indicator function.

This procedure can be used to control for the type-I error.

##### Parametric bootstrap procedure

The second resampling-based method is the parametric bootstrap procedure, which yields an asymptotic reference distribution. This procedure makes it possible to control for type-I error with fewer assumptions [21]. This procedure is summarized in the following algorithm [6]:

1. Apply the minimum *p*-value procedure to the original data for the *K* transformations considered. We note *p*_*min*_ the realization of the minimum of the *p*-value;
2. Fit the model under the null hypothesis, using the observed data, and obtain $\boldsymbol {\hat {\gamma }}$, the maximum likelihood estimate (MLE) of ***γ***;
3. Generate a new outcome $Y_{i}^{*}$ for each subject from the probability measure defined under $\mathscr {H}_{0,k}$.
4. Repeat this for all the subjects to obtain a sample denoted $s^{*}=\{Y^{*}_{i},\mathbf {Z_{i}},X_{i}\}$
5. Generate *B* new datasets $s_{b}^{*}, b=1,\hdots,B$ by repeating step 3 *B* times ;
6. For each new dataset, apply the minimum *p*-value procedure for the transformation considered. We note $p^{*b}_{\text {min}}$ the smallest *p*-value for each new dataset.
7. The *p*-value is then approximated by:
  $$\widehat{p^{minP}}=\frac{1}{B}\sum_{b=1}^{B}I_{\left\{p_{\text{min}}^{*b} < p_{\text{min}}\right\}}.$$

### Codings

We now provide some examples of available transformations in the CPMCGLM package.

#### Dichotomous coding

Dichotomous coding is often used in clinical and psychological research, either to facilitate interpretation, or because a threshold effect is suspected. In regression models with multiple explanatory variables, it may be seen as easier to interpret the regression coefficient for a binary variable than to understand a one-unit change in the continuous variable. In this context, dichotomous transformations of the variable of interest *X* are defined as:

$$X(k)= \left\{ \begin{array}{lll} 1& \text{if} & X\geq c_{k} ;\\ 0& \text{if} & X< c_{k},\\ \end{array} \right. $$

where *c*_*k*_ denotes the cutoff value for the transformation *k* (1≤*k*≤*K*).

In this R package, the dicho argument of the CPMCGLM() function allows the definition of desired cutoff points based on quantiles in a vector. An example of the dicho argument is provided below:


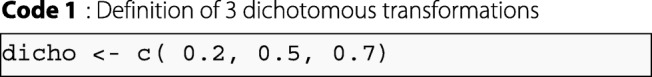


In this example, the user wants to try three dichotomous transformations of the variable of interest. For the first transformation, the cutoff point is the second decile; for the second, it is the median, and for the third, the seventh decile. The user can also opt to use our quantile-based method. The choice of this method leads to use of the nb.dicho argument. This argument makes it possible to use a quantile-based method, by entering the desired number of transformations. If the user asks for three transformations, the program uses the quartiles as cutoff points. If two transformations are requested, the program uses the terciles, and so on. This argument is also defined as follows.


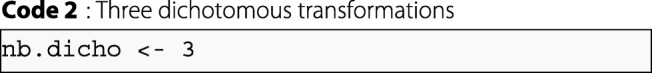


It is important to note that only one of these arguments (dicho and nb.dicho) can be used in a given CPMCGLM()function.

#### Coding with more than two classes

In epidemiology, it is usual to create several categories, often four or five. These transformations into categorical variables are defined as follows:

$$X(k)= \left\{ \begin{array}{lll} m-1& \text{if} & X\geq c_{k^{m-2}} ;\\ \vdots & & \vdots \\ j& \text{if} & c_{k^{j}} > X \geq c_{k^{j-1}} ;\\ \vdots & & \vdots \\ 0& \text{if} & X< c_{k^{0}},\\ \end{array} \right. $$

where $c_{k^{j}}\phantom {\dot {i}\!}$ denotes the *j*^*th*^ cutoff point (0≤*j*≤*m*−2), for the transformation *k* (1≤*k*≤*K*).

The categ argument of the CPMCGLM() function allows the user to define the desired set of cutoff points using quantiles. This argument must take the form of a matrix, with a number of columns matching the maximum number of cutoff points used in almost all transformations, and a number of rows corresponding to the number of transformations tried. An example of this argument definition is presented below:


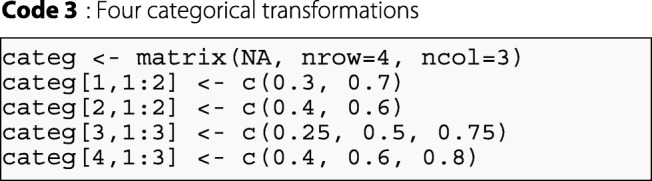


In this example, the user will realize four transformations. Two involve transformation into three classes, and two into four classes. It is important to note that binary transformations could not be defined here. The maximum number of cutoff points used in almost all transformations is three. The matrix therefore has the following dimensions: (4×3). For the first transformation, we will define a transformation into a three-class categorical variable with the third and seventh deciles as cut-points, and so on for the other transformations.

The user could also use a quantile-based method to define the transformations. In this case, the user would need to define the number of categorical transformations in the nb.categ argument. If two transformations are requested, then this method will create a two-class categorical variable using the terciles as cutoff points, and a three-class categorical variable using the quartiles as cutoff points. If the user asks for three transformations, the first and second transformations remain the same, and the program creates another categorical variable with four classes based on the quintiles, and so on. For four transformations, the argument is defined in R as follows:


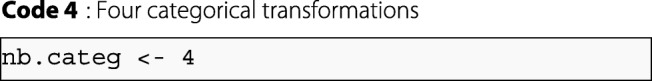


However, users may also wish to define their own set of thresholds. For this reason, the function also includes the argument cutpoint, which can be defined on the basis of true values for the transformations desired. This argument is a matrix, defined as the argument categ. The difference between this argument and that described above is that it is possible to define dichotomous transformations for this argument and quantiles are not used.


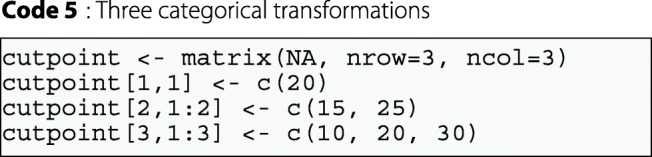


#### Box-Cox transformation

Other transformations are also used, including Box-Cox transformations in particular, defined as follows [22]:

$$X(k)= \left\{ \begin{array}{lll} \lambda_{k}^{-1}(X^{\lambda_{k}}-1) & \text{if} &\lambda_{k} > 0 \\ \log{X}& \text{if} & \lambda_{k} =0,\\ \end{array} \right. $$

This family of transformations incorporates many traditional transformations:

- *λ*_*k*_ = 1.00: no transformation needed; produces results identical to original data
- *λ*_*k*_ = 0.50: square root transformation
- *λ*_*k*_ = 0.33: cube root transformation
- *λ*_*k*_ = 0.25: fourth root transformation
- *λ*_*k*_ = 0.00: natural log transformation
- *λ*_*k*_ = -0.50: reciprocal square root transformation
- *λ*_*k*_ = -1.00: reciprocal (inverse) transformation

The boxcox argument is used to define Box-Cox transformations. This argument is a vector, and the values of its elements denote the desired *λ*_*k*_. An example of the boxcox argument for a reciprocal transformation, a natural log transformation, and a square root transformation is provided below:


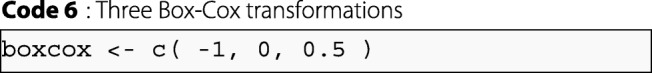


#### Fractional polynomial transformation

Royston et al. showed that traditional methods for analyzing continuous or ordinal risk factors based on categorization or linear models could be improved [23, 24]. They proposed an approach based on fractional polynomial transformation. Let us consider generalized linear models with canonical parameters defined as follows:

$$\theta_{i}(X,Z)=\boldsymbol{\gamma} \mathbf{Z_{i}}+ \beta \mathbf{X_{i}}, \ \ 1 \le i \le n;$$

where $\mathbf {Z_{i}}=\left (1,Z_{i}^{1},\hdots,Z_{i}^{q-1}\right), \boldsymbol {\gamma }=(\gamma _{0},\hdots,\gamma _{q-1})^{T}$ is a vector of *q* regression coefficients, and *β* is the coefficient associated with the *X*_*i*_ variable.

Consider the arbitrary powers *a*_1_≤…≤*a*_*j*_≤…≤ *a*_*m*_, with 1≤*j*≤*m*, and *a*_0_=0.

If the random variable *X* is positive, i.e. ∀*i*∈{1,…,*n*},*X*_*i*_>0, then the fractional polynomial transformation is defined as:

$$\theta_{i}^{m}(X,Z,\xi,a)=\boldsymbol{\gamma} \mathbf{Z_{i}}+\sum_{j=0}^{m}\xi_{j}H_{j}(X_{i}),$$

where for 0≤*j*≤*m* *ξ*_*j*_ is the coefficient associated with the fractional polynomial transformation:

$$H_{j}(X_{i})= \left\{ \begin{array}{ll} X_{i}^{(a_{j})} & \text{ if \(a_{j} \neq a_{j-1}\)} \\ H_{j-1}(X_{i})ln(X_{i}) & \text{ if \(a_{j} = a_{j-1}\) } \end{array} \right. $$

where *H*_0_(*X*_*i*_)=1.

However, if non-positive values of *X* can occur, a preliminary transformation of *X* to ensure positivity is required. The solution proposed by Royston and Altman is to choose a non-zero origin *ζ*<*X*_*i*_ and to rewrite the canonical parameter of the model for fractional polynomial transformation as follows:

$$\theta_{i}^{m}(X,Z,\xi,a)=\boldsymbol{\gamma} \mathbf{Z_{i}}+\sum_{j=0}^{m}\xi_{j}H_{j}(X_{i}-\zeta),$$

*ζ* is set to the lower limit of the rounding interval of samples values for the variable of interest.

Royston and Altman suggested using *m* powers from a predefined set $\mathscr {P}$ [25]:

$$\begin{array}{*{20}l} \mathscr{P}= \{ -\text{max}(3,m);\hdots ;-2;-1;-0.5;0;0.5;1;2; \hdots;\text{max}(3,m) \}. \end{array} $$

The FP argument is used to define these transformations. This argument is a matrix. The number of rows correspond to the number of transformations tested, and the number of columns is the maximum number of degrees tested for a single transformation. An example of the FP argument:


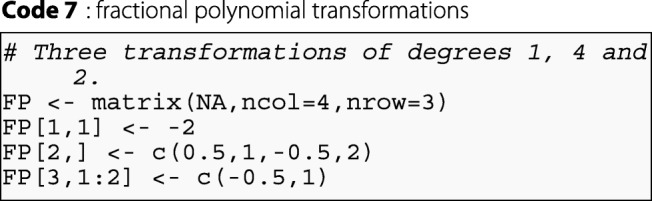


In this example, the user performs three transformations of the variable of interest. The first is a fractional polynomial transformation with one degree and a power of − 2. The second transformation is a fractional polynomial transformation with four degrees and powers of 0.5,1,−0.5, and 2. The third transformation is a fractional polynomial transformation with two degrees and powers of − 0.5, and 1.

### Motivating example

We revisited the example presented in the article of Liquet and Commenges in 2001 based on the PAQUID database [11], to illustrate the use of the CPMCGLM package, in the context of logistic regression.

#### PAQUID database

PAQUID is a longitudinal, prospective study of individuals aged at least 65 years on December 31, 1987 living in the community in France. These residents live in two administrative areas in southwestern France. This elderly population-based cohort of 3111 community residents aimed to identify the risk factors for cognitive decline, dementia, and Alzheimer’s disease. The data were obtained in a nested case-control study of 311 subjects from this cohort (33 subject with dementia and 278 controls).

#### Scientific aims

The analysis focused on the influence of HDL(high-density lipoprotein)-cholesterol on the risk of dementia. We considered the variables age, sex, education level, and wine consumption as adjustment variables. Bonarek *et al* initially considered HDL-cholesterol as a continuous variable [26]. Subsequently, to facilitate clinical interpretation, they decided to transform this variable into a categorical variable with different thresholds, and different numbers of classes. This strategy implied the use of multiple models, and multiple testing. A correction of type-I error taking into account the various transformations performed was therefore required to identify the best association between dementia and HDL-cholesterol.

#### Methods

We applied the various types of correction method described in this article to correct the type-I error rate in the model defined above. These corrections are easy to apply with the CPMCGLM package. The following syntax provided the desired results for one categorical coding, three binary codings, one Box-Cox transformation with *λ*=0, and one fractional polynomial transformation with two degrees and powers of -0.5, and 1:


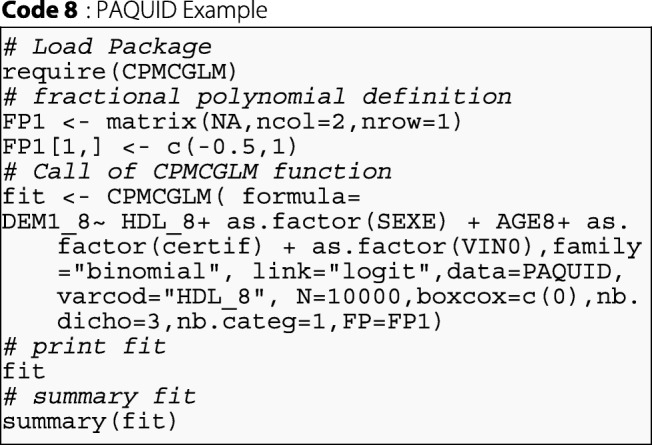


By using the "dicho", and "categ" arguments, the function could also be used as follows, for exactly the same analysis:


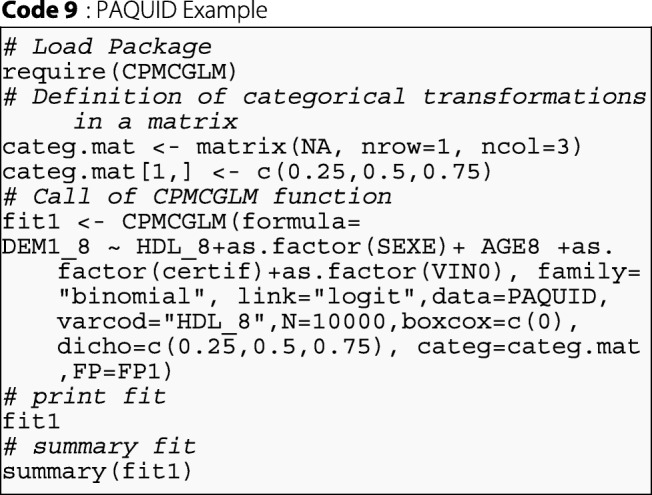


#### Results

In R software, the results obtained with the CPMCGLM package described above are summarized as follows:


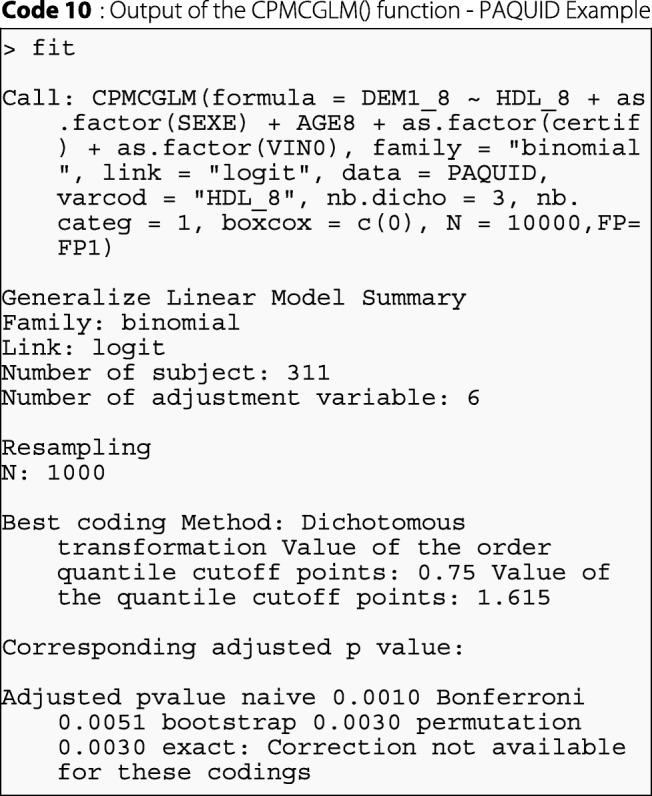


We can also use the summary function for the main results, which are described as follows for this specific result:


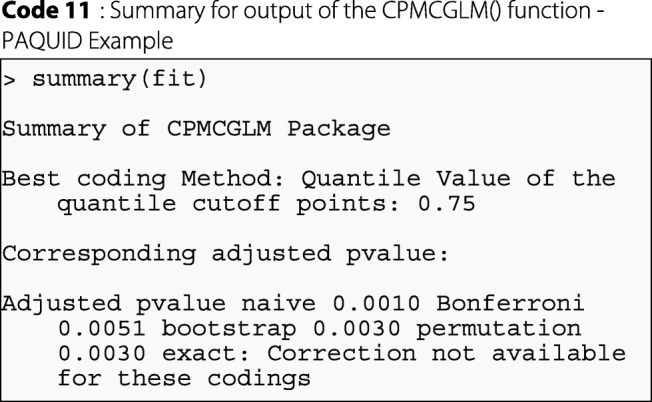


As we can see, for this example, the best coding was obtained for the logistic regression with dichotomous coding of the HDL-cholesterol variable. The cutoff point retained for this variable was the third quartile. Exact correction was not available for this application, due to the use of transformation into categorical variables with more than two classes. Resampling methods gave similar results, and both the resampling methods tested were more powerful than Bonferroni correction. In conclusion, the correction of type-I error is required. Naive correction is not satisfactory, and resampling methods seem to give the best results for *p*-value correction in this example.

## Conclusion

We present here CPMCGLM, an R package providing efficient methods for the correction of type-I error rate in the context of generalized linear models. This is the only available package in R providing such methods applied to this context. We are currently working on the generalization of these methods to proportional hazard models, which we will make available as soon as possible in the CPMCGLM package.

In practice, it is important to correct the multiplicity on all the codings that have been tested. Indeed, if this is not done, the type-I error is not controlled, and then it is possible to obtain some false positive results.

To conclude, this package is designed to help researchers who work principally in epidemiology to analyze with riguor their data in the context of optimal cutoff point determination.

## Availability and requirements

**Project name:** CPMCGLM

**Project home page:** https://cran.r-project.org/web/packages/CPMCGLM/index.html

**Operating system(s):** Platform independent

**Programming language:** R

**Other requirements:** R 2.10.0 or above

**License:** GPL-2

**Any restrictions to use by non-academics:** none

## Abbreviations

FP: Fractional polynomial
GLM: Generalized linear model
HDL: High-density lipoprotein
MLE: Maximum likelihood estimate
PAQUID: Personnes agées QUID
